# Supplementary material for: The association between social factors and COVID-19 protective behaviors and depression and stress among midwestern US college students
Source: PLoS One. 2022 Dec 19;17(12):e0279340. doi: 10.1371/journal.pone.0279340 (PMC9762587; doi:10.1371/journal.pone.0279340)
Supplement: Attachment — Submitted filename: Manuscript_commented.docx [file pone.0279340.s002.docx]

Social factors associated with college students' depressionTh and stress during the COVID-19 pandemic: A cross-sectional study

Edlin Garcia Colato^1*^, Christina Ludema^2^, Molly Rosenberg^2^, Sina Kianersi^2^, Maya Luetke^3^, Chen Chen^2^, and Jonathan T Macy^1^

^1^Indiana University School of Public Health Bloomington, Department of Applied Health Science, Bloomington, Indiana, United States of America

^2^Indiana University School of Public Health Bloomington, Department of Epidemiology and Biostatistics, Bloomington, Indiana, United States of America

^3^University of Minnesota, Institute for Social Research and Data Innovation, Minneapolis, Minnesota, United States of America

*Correspondence concerning this article should be addressed to Edlin Garcia Colato.

Email: eggarcia@iu.edu.

**Abstract**

The aim of this cross-sectional study was to examine the relationship between social factors and COVID-19 protective behaviors and two outcomes: depressive and perceived stress symptoms. In September 2020, 1,064 randomly selected undergraduate students from a large Midwestern University completed an online survey and provided information on demographics, social activities, COVID-19 protective behaviors (i.e., avoiding social events and staying home from work and school), and mental health symptoms. Mental health symptoms were measured using the Center for Epidemiological Studies Depression-10 (CES-D-10) questionnaire for depression and the Perceived Stress Scale-10 (PSS-10) for stress symptoms. Logistic and linear regression analyses were performed on the CES-D-10 and PSS-10 scores. Males were significantly less likely to report depressive symptoms and stress symptoms compared to females. ‘Hanging out’ with more people while drinking alcohol was associated with lower depressive symptoms and lower stress symptoms. Staying home from work or school “very often” was associated with higher stress symptoms, compared with “never/rarely” staying home from work/school. Having a job with in-person interaction was also associated with increased stress. These findings suggest that lack of social engagement was associated with depression and stress symptoms among college students during the COVID-19 pandemic. Planning social activities that align with recommended safety precautions, as well as meet students’ social needs, should be an important priority for higher education institutions.

Keywords: COVID-19, Depressive symptoms, Perceived stress symptoms, Mental health

**Introduction**

**Background**

With the emergence of the Coronavirus Disease 2019 (COVID-19) pandemic, the public received stay-at-home orders and recommendations to wear masks, reduce participation in large social events, and maintain six feet of physical distance, among other protective behaviors to reduce risk of severe acute respiratory syndrome coronavirus 2 (SARS-CoV-2) transmission. In addition to the risk to physical health posed by the virus, approximately 80% of adults reported that the COVID-19 pandemic was a significant source of stress [1]. Young adults in college are a particularly vulnerable group. During college, young adults may experience a variety of mental health symptoms, such as those related to depression and stress, as well as experiment with substances such as e-cigarettes/vaping and alcohol [2]. Mild to severe symptoms of depression are highest among 18 to 29-year-olds (21%) and females (21.8%) [3]. Stressors for college students may involve academic pressures, financial worries, and family obligations; with the COVID-19 pandemic adding to that stress. In a study of Australian university students conducted before the COVID-19 pandemic, field of study, hours of study per week, hours of paid work per week, and number of hours caring for family per week were found to be associated with increased severity of depression and stress [4]. Many undergraduates are employed [5, 6] to help afford courses, housing, or other living expenses, which could also contribute to stress. Acute stressors are a part of everyday life, but chronic stress can lead to more serious conditions such as anxiety or depression [7]. Significant increases in perceived stress and depression were found among undergraduate students at a large Northeastern US university in Spring 2020 during the start of the pandemic [8].

College student life is also characterized by the consumption of alcohol. Drinking alcohol is viewed as a social activity, influenced by time spent with peers [9]. Alcohol is the most frequently used substance, reportedly consumed by 53.5% of full-time U.S. college students ages 18-22 [10]. During the COVID-19 lockdown, Belgian college students were found to have significantly reduced their alcohol use [11]. Similarly, e-cigarette use, which has also been on the rise among young adults, saw a decrease in use during the beginning of the COVID-19 pandemic, yet some young adults changed their overall substance use habits [12, 13]. These reductions in substance use could be suggestive of fewer opportunities for social gatherings among students as a result of COVID-19 prevention policies.

The COVID-19 pandemic has brought new challenges to the efforts of keeping college students safe and healthy. There has been limited research related to past epidemics and their effects on mental health [14], making it difficult to discern the extent of the impact of the COVID-19 pandemic on college students’ psychological health [15]. Studies focused on college student mental health during the earlier days of the COVID-19 pandemic found that social support was associated with better psychological health [16]. Compared to other age groups, young adults were found to be at highest risk of loneliness as a result of the pandemic [17]. However, it is unknown if social factors, staying home from work or school, or avoiding social events were associated with symptoms of depression and stress among young adults in college during the first semester back at the university since the start of the COVID-19 pandemic. A particular challenge is finding the right balance between having people engage in COVID-19 protective behaviors, but without it leading to negative consequences for their mental health [15]. A recent study involving college students from seven U.S. states found that those between the ages 18 to 24, women, those with fair/poor general health, those who had eight or more daily hours of screen time, or those who knew someone infected with COVID-19 all had higher levels of psychological impact [18]. In a study of Chinese college students, academic workload, separation from school, and fears of infection were found to have negative effects perceived stress [19].

**Objectives**

COVID-19 mental health studies have primarily examined the relationships between behaviors and mental health symptoms during the beginning months of the pandemic [17-23]. Few have assessed US college students’ mental health in Fall 2020 during the COVID-19 pandemic [24, 25]. The primary objective of the current study was to examine the relationship between social factors and COVID-19 protective behaviors and two measures of mental health status: depressive and stress symptoms. We used the Strengthening the Reporting of Observational Studies in Epidemiology (STROBE) for cross-sectional studies checklist.

**Methods**

**Study Design**

A cross-sectional study design was used to identify the prevalence of depressive and stress symptoms among undergraduates in September 2020 (beginning of the fall semester). All data were retrieved from the online self-reported baseline survey collected through REDCap (Research Electronic Data Capture) [26, 27]. Participants received up to $30 for completing the study activities. This investigation was performed in accordance with the ethical standards laid down in the 1964 Declaration of Helsinki and its later amendments. The study design was reviewed by an appropriate ethical committee and received Institutional Review Board approval (Protocol #2008293852). Informed consent of the participants was obtained after the nature of the procedures had been fully explained.

**Setting**

Undergraduate students at a large Midwestern university were randomly sampled to participate in a SARS-CoV-2 antibody study during the fall 2020 semester [28]. Participants completed baseline surveys between September 8 and 30, 2020.

**Participants**

The Office of the Vice Provost for Undergraduate Education generated a random list of undergraduates to be representative of the undergraduate student population (~32,620). To be eligible for the antibody study, participants had to be 1) enrolled as an undergraduate student, 2) aged 18 years or older, and 3) residing in the same county where the university is located [28].

**Variables**

**Outcome (1): Depression (CES-D-10)**

The 10-item Center for Epidemiological Studies Depression (CES-D-10) previously validated questionnaire was used to capture self-reported symptoms of depression [29-31]. Responses to the 10 questions (0 = “rarely or none of the time (less than 1 day)” to 3 = “all of the time (5-7 days)”) were summed. Possible total scores ranged from 0 to 30. The cut-off point used was 10, which was shown in the validation to be equivalent to the cut-off value used in the original CES-D-20 questionnaire [31]. Summed scores below 10 were categorized as 0 = “no significant depressive symptoms” (reference group) and the sum of scores equaling 10 or above were categorized as 1 = “significant depressive symptoms” and were entered into the logistic regression model as a binary outcome. Cronbach’s alpha for the CES-D-10 scale was 0.84.

**Outcome (2): Stress (PSS-10)**

Symptoms related to stress were captured using the 10-item Perceived Stress Scale (PSS-10), a reliable and previously validated instrument [32]. All ten questions had a 5-point scale, from 0 = “never” up to 4 = “very often,” four of which were positively stated and therefore reverse coded before scores were summed. Scores ranging from 0 to 13 were coded as 0 = “low stress”; scores 14 to 26 were coded as 2 = “moderate stress”; and scores between 27 and 40 were coded as 3 = “high stress” for descriptive purposes [33]. Higher scores are associated with increased perceived stress. The PSS-10 scores are categorized for descriptive purposes and do not translate into clinical diagnostic significance [34]. For analyses, summed scores for stress symptoms were entered into a linear regression model as the outcome variable. Cronbach’s alpha for the PSS-10 was 0.85.

**Covariates**

**Sex.** On the baseline survey, participants were asked, “*What sex were you assigned at birth, on your original birth certificate?*” with options “male*”* and “female.”

**Academic factors.** Participants reported total overall credit hours and total in-person credit hours using a drop-down menu ranging from zero to 30; both were recorded as continuous variables.

**Social factors.** Participants were asked whether they had a job or internship that involved in-person interactions (1 = yes vs. 0 = no). Participants were also asked whether they “*ever used any of the following inhaled tobacco products before today? Cigarettes, e-cigarettes, other inhaled products, and none of the above”* and were asked to mark all that apply. Past 30-day e-cigarette use was categorized into 0 = “none/zero days*,*” 1 = “1-5 day(s),” and 2 = “>5 days” from responses ranging from 1 to 30 for the “*During the last 30 days, on how many days did you use e-cigarettes?*” question. Related to alcohol drinking behaviors, participants were asked to enter a numerical value of the number (ranging from 0 to 1,000) of persons/partners they typically ‘hang out’ with while drinking alcohol. Non-alcohol drinkers were recoded with zero for the number of drinking partners. A separate group that was restricted to only the participants that reported drinking alcohol was also created for the reported number of drinking partners/ number of people the participants ‘hang out’ with.

**COVID-19 Protective Behaviors.** Participants were asked if they practiced a series of protective behaviors in the past 7 days to prevent infection of COVID-19. The two included in this study are: how often they “*avoided a social event I wanted to attend”* followed by how often they *“stayed at home from work/school”* with options 1 = “always,” 2 = “very often,” 3 = “sometimes,” 4 = “rarely,” and 5 = “never” for both questions. The responses to both questions were reverse coded, and “rarely” and “never” were collapsed.

**Data Analysis**

The following models were conducted: 1) unadjusted and sex-adjusted logistic regression models for the relationship between each of the four social and behavioral variables and depressive symptoms, 2) unadjusted and sex-adjusted linear regression models to test for the association between the four social and behavioral variables and stress symptoms. Sex is associated with both prevalence and severity of reported depressive symptoms [3] and differences in stress levels [35]. Therefore, sex was controlled for in the adjusted models. The data analyses were generated using Stata software, version 16.1 (College Station, Texas).

**Results**

**Participants**

Of the 7,499 undergraduate students randomly sampled to participate in the COVID-19 antibody study, 3,430 were ineligible because they were not living in the same county as the university or not enrolled as an undergraduate student. Of the 4,069 eligible individuals, 1,397 (34.4%) provided voluntary electronic consent. One hundred thirty-three discontinued their involvement in the study by not answering any of the survey questions and were therefore excluded from this analysis.

For the mental health symptoms outcomes, 108 of the 1,264 participants had missing values for the CES-D-10 and PSS-10 questions and were removed. Of the 1,156 with complete mental health symptoms data, 91 (7.9%) participants had missing self-reported background information: age (missing 68), sex at birth (missing 1), school year (missing 3), credit hours (missing 1), in person credit hours (missing 11), job with in-person interaction (missing 2), number of alcohol drinking partners (missing 3), and COVID-19 protective behaviors (missing 2) and were removed. One participant entered zero for the total credits enrolled in and was also removed, yielding n = 1,064 for the final sample.

**Descriptive data**

Table 1 summarizes the socio-demographic characteristics of the participants by both depressive and stress symptoms. Most respondents were female, White, non-Hispanic/Latinx, had none/zero day(s) of past 30-day e-cigarette use, and did not have a job or internship with in-person interaction. Of the 721 students that reported drinking alcohol, 705 (97.8%; not shown in table) reported ‘hanging out’ with people while drinking. Of the students with an in-person facing job or internship, the majority (219; 74.0%) were females (not shown in tables).

| **Table 1.** **Depressive Symptoms and Perceived Stress Scores in a Sample of Undergraduate Students from a Midwestern University** | | | | | | | | |
| --- | --- | --- | --- | --- | --- | --- | --- | --- |
|  |  | Depressive Symptoms | | Perceived Stress Score | | | | |
|  | N | No (%) | Yes (%) | Mean (SD) | Range | Low Stress  n (%) | Moderate  Stress n (%) | High Stress  n (%) |
| **All** | 1,064 | 617 (58.0) | 447 (42.0) | 18.28 (6.52) | 0-38 | 254 (23.9) | 705 (66.3) | 105 (9.9) |
| **Age,** mean (SD) | 20.03 (2.49) | 20.11 (2.88) | 19.91 (1.82) |  |  | 20.30 (3.70) | 19.96 (2.00) | 19.83 (1.68) |
| **Sex** |  |  |  |  |  |  |  |  |
| Female | 686 (64.5) | 350 (56.7) | 336 (75.2) | 19.41 (6.43) | 2-38 | 130 (51.2) | 471 (66.8) | 85 (80.9) |
| Male | 378 (35.5) | 267 (43.3) | 111 (24.8) | 16.22 (6.16) | 0-35 | 124 (48.8) | 234 (33.2) | 20 (19.0) |
| **Gender** |  |  |  |  |  |  |  |  |
| Female | 675 (63.5) | 349 (56.7) | 326 (72.9) | 19.33 (6.42) | 2-38 | 130 (51.2) | 465 (66.0) | 80 (76.2) |
| Male | 375 (35.3) | 266 (43.2) | 109 (24.4) | 16.19 (6.14) | 0-35 | 124 (48.8) | 232 (32.9) | 19 (18.1) |
| Transgender | 4 (0.4) | 1 (0.2) | 3 (0.7) | 22.50 (5.51) | 17-29 | 0 | 3 (0.4) | 1 (0.9) |
| None of the above | 9 (0.8) | 0 | 9 (2.0) | 25.67 (5.36) | 16-32 | 0 | 4 (0.6) | 5 (4.8) |
| **Race** |  |  |  |  |  |  |  |  |
| African American/Black | 17 (1.6) | 11 (1.8) | 6 (1.3) | 16.00 (8.28) | 5-30 | 9 (3.5) | 6 (0.8) | 2 (1.9) |
| Asian | 77 (7.2) | 47 (7.6) | 30 (6.7) | 17.99 (6.20) | 5-35 | 18 (7.1) | 54 (7.7) | 5 (4.8) |
| Multiracial | 53 (5.0) | 33 (5.3) | 20 (4.5) | 17.40 (7.03) | 0-35 | 15 (5.9) | 33 (4.7) | 5 (4.8) |
| Other | 50 (4.7) | 23 (3.7) | 27 (6.0) | 19.22 (5.57) | 3-29 | 6 (2.4) | 39 (5.5) | 5 (4.8) |
| White | 867 (81.5) | 503 (81.5) | 364 (81.4) | 18.35 (6.52) | 0-38 | 206 (81.1) | 573 (81.3) | 88 (83.8) |
| **Ethnicity** |  |  |  |  |  |  |  |  |
| Hispanic/Latinx | 85 (8.0) | 47 (7.6) | 38 (8.5) | 18.88 (5.47) | 6-35 | 10 (3.9) | 68 (9.6) | 7 (6.7) |
| Non-Hispanic/Latinx | 979 (92.0) | 570 (92.4) | 409 (91.5) | 18.23 (6.59) | 0-38 | 244 (96.1) | 637 (90.4) | 98 (93.3) |
| **School Year** |  |  |  |  |  |  |  |  |
| First | 239 (22.5) | 146 (23.7) | 93 (20.8) | 17.48 (6.67) | 0-37 | 67 (26.4) | 151 (21.4) | 21 (20.0) |
| Second | 229 (21.5) | 119 (19.3) | 110 (24.6) | 19.37 (6.52) | 3-35 | 45 (17.7) | 154 (21.8) | 30 (28.6) |
| Third | 260 (24.4) | 151 (24.5) | 109 (24.4) | 18.53 (6.51) | 0-35 | 59 (23.2) | 176 (25.0) | 25 (23.8) |
| Fourth | 309 (29.0) | 187 (30.3) | 122 (27.3) | 17.87 (6.39) | 1-38 | 79 (31.1) | 204 (28.9) | 26 (24.8) |
| Fifth **+** | 27 (2.5) | 14 (2.3) | 13 (2.9) | 18.37 (5.54) | 11-31 | 4 (1.6) | 20 (2.8) | 3 (2.9) |
| **Total Credit hours** mean (SD) | 15.19 (2.17) | 15.17 (2.08) | 15.22 (2.29) |  |  | 15.20 (2.17) | 15.17 (2.17) | 15.28 (2.22) |
| **In-person Credit hours** mean (SD) | 2.99 (2.97) | 3.06 (3.01) | 2.88 (2.91) |  |  | 3.00 (2.90) | 3.02 (3.02) | 2.74 (2.82) |
| **Job or internship†** |  |  |  |  |  |  |  |  |
| No | 768 (72.2) | 464 (75.2) | 304 (68.0) | 17.72 (6.50) | 0-38 | 201 (79.1) | 50 (71.1) | 66 (62.9) |
| Yes | 296 (27.8) | 153 (24.8) | 143 (32.0) | 19.75 (6.34) | 2-35 | 53 (20.9) | 204 (28.9) | 39 (37.1) |
| **Past 30-day E-cigarette Use** |  |  |  |  |  |  |  |  |
| None/zero day(s) | 762 (71.6) | 450 (72.9) | 312 (69.8) | 18.15 (6.62) | 0-38 | 187 (73.6) | 500 (70.9) | 75 (71.4) |
| 1-5 days | 101 (9.5) | 56 (9.1) | 45 (10.1) | 18.37 (6.00) | 6-30 | 21 (8.3) | 70 (9.9) | 10 (9.5) |
| > 5 days | 201 (18.9) | 111 (18.0) | 90 (20.1) | 18.75 (6.36) | 3-37 | 46 (18.1) | 135 (19.1) | 20 (19.0) |
| **Number of People Hanging out with while Drinking Alcohol**, mean (SD) | 3.82 (4.78) | 4.44 (5.41) | 2.97 (3.58) |  |  | 4.12 (4.64) | 3.90 (5.00) | 2.54 (3.19) |
| **Avoided Social Events interested in attending**‡ |  |  |  |  |  |  |  |  |
| Always | 316 (29.7) | 169 (27.4) | 147 (32.9) | 19.02 (6.72) | 0-37 | 68 (26.8) | 206 (29.2) | 42 (40.0) |
| Very Often | 283 (26.6) | 156 (25.3) | 127 (28.4) | 18.30 (6.32) | 3-35 | 68 (26.8) | 191 (27.1) | 24 (22.9) |
| Sometimes | 275 (25.8) | 168 (27.2) | 107 (23.9) | 18.01 (6.43) | 0-38 | 66 (26.0) | 187 (26.5) | 22 (20.9) |
| Never/Rarely | 190 (17.9) | 124 (20.1) | 66 (14.8) | 17.41 (6.49) | 2-35 | 52 (20.5) | 121 (17.2) | 17 (16.2) |
| **Stayed Home From Work/School**‡ |  |  |  |  |  |  |  |  |
| Always | 266 (25.0) | 157 (25.4) | 109 (24.4) | 18.37 (6.92) | 0-38 | 62 (24.4) | 175 (24.8) | 29 (27.6) |
| Very Often | 198 (18.6) | 105 (17.0) | 93 (20.8) | 19.29 (6.31) | 0-35 | 36 (14.2) | 137 (19.4) | 25 (23.8) |
| Sometimes | 170 (16.0) | 97 (15.7) | 73 (16.3) | 18.13 (6.16) | 4-35 | 38 (15.0) | 117 (16.6) | 15 (14.3) |
| Never/Rarely | 430 (40.4) | 258 (41.8) | 172 (38.5) | 17.82 (6.46) | 2-35 | 118 (46.5) | 276 (39.1) | 36 (34.3) |
| *Note.* †with in-person interaction; ‡Measure taken in the last 7 days to prevent infection from COVID-19; CES-D-10 <10 = no; CES-D-10 >10 = yes; PSS-10 0-13 = low stress; PSS-10 14-26 = moderate stress; PSS-10 27-40 = high stress | | | | | | | | |

**Outcome data**

Four hundred forty-seven (42.0%) participants reported having significant depressive symptoms. Based on the PSS-10, most of the respondents (705; 66.3%) experienced moderate stress symptoms, followed by 23.9% with low stress and 9.9% with high stress. The mean score on the PSS-10 was 18.28 (SD 6.52) and ranged from zero to 38.

**Main results**

**Social/behavioral predictors of depressive symptoms in the unadjusted analyses**

As shown in Table 2, males were less likely than females to report significant depressive symptoms. Students with an in-person facing job or internship were more likely to report significant depressive symptoms than participants without an in-person facing job or internship. However, the number of total credit hours and in-person credit hours were not associated with increased depressive symptoms.

| **Table 2. Results for Logistic Regression for Depressive Symptoms (CES-D-10) and** **Linear Regression for Stress Symptoms (PSS) by Predictors (95% CI)** | | | | | | |
| --- | --- | --- | --- | --- | --- | --- |
|  | Depressive Symptoms | | | | Stress Symptoms | |
|  | Unadjusted OR | | Adjusted^a^ OR | Unadjusted $\beta$ | | Adjusted^a^$\beta$ |
| Sex |  |  |  |  |  |  |
| Female | Ref | | - - | Ref | | - - |
| Male | **0.43 (0.33, 0.56)***** | | - - | **-3.19 (-3.99, -2.40)***** | | - - |
| Total credit hours | 1.01 (0.96, 1.07) *ns* | | 1.01 (0.95, 1.07) *ns* | 0.002 (-0.18, 0.18) *ns* | | -0.02 (-0.19, 0.16) *ns* |
| Credit hours in person | 0.98 (0.94, 1.02) *ns* | | 0.98 (0.94, 1.02) *ns* | -0.07 (-0.20, 0.06) *ns* | | -0.07 (-0.19, 0.06) *ns* |
| Job or internship† |  |  |  |  |  |  |
| No | Ref | | Ref | Ref | | Ref |
| Yes | **1.43 (1.09, 1.87)*** | | 1.29 (0.98,1.71) *ns* | **2.03 (1.16, 2.90)**** | | **1.63 (0.78, 2.49)***** |
| E-cigarette Use Past 30-days |  | |  |  |  |  |
| None/0 days | Ref | | Ref | Ref | | Ref |
| 1-5 days | 1.16 (0.76, 1.76) *ns* | | 1.22 (0.80, 1.88) *ns* | 0.22 (-1.13, 1.57) *ns* | | 0.40 (-0.91, 1.72) *ns* |
| > 5 days | 1.17 (0.85, 1.60) *ns* | | 1.29 (0.94, 1.78) *ns* | 0.60 (-0.41, 1.61) *ns* | | 0.95 (-0.03, 1.94) *ns* |
| Number of People Hanging out with while Drinking Alcohol | **0.92 (0.89, 0.95)***** | | **0.92 (0.89, 0.95)***** | **-0.13 (-0.22, -0.06)**** | | **-0.13 (-0.21, -0.05)**** |
| Avoided Social Events Interested in Attending |  | |  |  |  |  |
| Never/Rarely | Ref | | Ref | Ref | |  |
| Sometimes | 1.20 (0.81, 1.76) *ns* | | 1.09 (0.74, 1.62) *ns* | 0.60 (-0.60, 1.81) *ns* | | 0.23 (-0.95, 1.40) *ns* |
| Very often | **1.53 (1.05, 2.23)*** | | 1.38 (0.94, 2.03) *ns* | 0.89 (-0.31, 2.09) *ns* | | 0.44 (-0.73, 1.61) *ns* |
| Always | **1.63 (1.13, 2.37)*** | | 1.40 (0.96, 2.05) *ns* | **1.61 (0.44, 2.78)**** | | 0.95 (-0.21, 2.10) *ns* |
| Stayed Home from Work/School |  | |  |  |  |  |
| Never/Rarely | Ref | | Ref | Ref | | Ref |
| Sometimes | 1.13 (0.79, 1.62) *ns* | | 1.12 (0.78, 1.61) *ns* | 0.31 (-0.85, 1.46) *ns* | | 0.26 (-0.86, 1.38) *ns* |
| Very often | 1.33 (0.95, 1.86) *ns* | | 1.33 (0.94, 1.89) *ns* | **1.46 (0.37 2.56)**** | | **1.44 (0.37, 2.51)**** |
| Always | 1.04 (0.76, 1.42) *ns* | | 1.05 (0.77, 1.45) *ns* | 0.54 (-0.45, 1.54) *ns* | | 0.58 (-0.38, 1.55) *ns* |
| ^a^Adjusted for sex; †with in-person interaction; *ns* = not significant; *p-value <0.05; **<0.01; ***<0.001 | | | | | | |

Past 30-day e-cigarette use was also not found to be associated with depressive symptoms. For a one unit increase in the number of people ‘hanging out’ with while drinking alcohol, the odds of reporting significant depressive symptoms decreased. Similarly, among the restricted sample of students who reported drinking, for each one unit increase in the number of people ‘hanging out’ with while drinking alcohol, the odds of reporting significant depressive symptoms decreased. Those who avoided social events either “very often” and “always” had higher odds of reported significant depressive symptoms compared to those who reported “never/rarely” avoiding those social events.

**Social/behavioral Predictors of Depressive Symptoms in the Sex-Adjusted Analyses**

No significant relationship was found between the number of total credit hours and in-person credit hours and depressive symptoms, after adjusting for sex. However, sex largely accounted for the association between having an in-person facing job or internship and significant depressive symptoms. There was no longer a significant association between having an in-person facing job or internishp and depressive symptoms after adjusting for sex.

The relationship between past 30-day e-cigarette use and depressive symptoms remained relatively unchanged and still not significant, after controlling for sex. The significant association between the number of people the students ‘hung out’ with while drinking alcohol and depressive symptoms remained unchanged after controlling for sex in the model. Likewise, in the restricted sample of participants who reported drinking alcohol, the significant association between the number of people the students ‘hung out’ with while drinking alcohol and depressive symptoms was also identical after controlling for sex. However, both of the COVID-19 protective behaviors (avoiding social events and staying home from work and school) were not significantly associated with having significant depressive symptoms when controlling for sex in the models.

**Social/behavioral predictors of stress symptoms in the unadjusted analyses**

As shown in Table 2, males were significantly less likely to report stress symptoms compared to females. Number of total credit hours and in-person credit hours were not associated with increased stress symptoms. However, having a job or internship with in-person interaction was associated with reporting greater stress symptoms.

There was no statistically significant relationship between the past 30-day e-cigarette use and stress. Increased number of reported people ‘hanging out’ with while drinking alcohol was negatively associated with stress. Among the restricted sample of students who reported drinking, the increased number of reported people ‘hanging out’ with while drinking alcohol was similarly negatively associated with stress.

Among the COVID-19 protective behaviors, participants who “always” avoided social events reported greater stress symptoms compared to those who “never/rarely” attended social events. In addition, staying home from work/school “very often” was associated with reporting greater stress symptoms compared to “never/rarely” staying home from work/school.

**Social/behavioral predictors of stress symptoms in the sex-adjusted analyses**

After controlling for sex, there was no relationship between the number of total credit hours and in-person credit hours and stress symptoms. However, the association between having a job or internship with in-person interaction and increased stress remained significant. The relationship between the increased number of people the respondents ‘hung out’ with while drinking alcohol and increased stress symptoms also remained significant. Similarly, among the restricted sample of participants who reported drinking alcohol, the increased number of people the respondents ‘hung out’ with while drinking alcohol and increased stress was significant after controlling for sex. Lastly, the relationship between “always” staying home from work/school compared to “never/rarely” remained significant after controlling for sex in the model.

**Discussion**

**Key results**

In this study, we found that less than half of the undergraduate student participants reported depressive symptoms, and the majority reported moderate to high levels of stress symptoms in Fall 2020. We also found that key social and behavioral variables were associated with these negative health outcomes. Namely, these included having a job or internship with in-person interaction, the number of people students ‘hung out’ with while drinking alcohol, avoiding social events, and staying home from work or school. The relationship between the number of people students ‘hung out’ with while drinking alcohol and both depressive and stress symptoms persisted even after accounting for the expected and strong sex differences in our two mental health outcomes.

The main objective of this study was to test the relationships between social and COVID-19 protective behaviors and the two mental health outcomes: symptoms of depression and stress. The percentage of reported high stress scores in our sample (9.9%) was lower than the July/August 2020 PSS-10 scores from undergraduates at a Southeastern US university where 26.9% reported high stress [23]. Findings in this study showed that males were less likely to have depressive or increased stress symptoms compared to females, similar to previous findings [3, 34, 36]. On the other hand, total credit hours and in-person credit hours were not found to have a statistically significant association with either depressive or stress symptoms. This could be due to the added university safety features requiring students to wear masks and social distance, as well as the enhanced cleaning procedures and limited classroom capacity for in-person lectures, which may have helped prevent any significant stress. It should also be noted that undergraduates were considered full-time with an enrollment of at least 12 credit hours per semester. However, the University recommends that undergraduates complete approximately 15 to 16 credit hours during the fall and spring semesters to complete their degree in four years. Therefore, on average, students were enrolled in the recommended amount of 15 credit hours.

Participants who reported having a job or internship with in-person interaction reported significantly increased stress. Students employed or interning in a position that required interacting with people in-person were potentially presented with unique stressors brought on by balancing work responsibilities, as they also had to engage in COVID-19 protective behaviors.

Past 30-day e-cigarette use was not found to have a cross-sectional association with depressive symptoms. This finding does not align with the Obisesan, Mirbolouk [37] Behavioral Risk Factor Surveillance System data analysis of college students, in which increased e-cigarette use (occasional and daily versus never) was associated with reported depression. The difference in this finding might be due to the measurement tool used for depression. The prior study included a much larger cross-sectional sample and measured depression based on self-reported history of ever being diagnosed with depression, whereas our study used the CES-D-10 to identify depression.

For the COVID-19 protective behaviors, there was significant increased perceived stress among participants who reported they “very often” stayed home from work/school compared to those who “rarely/never” stayed home from work/school. However, this association was not observed for participants who reported “always” staying home. The undergraduates who reported staying home “very often” may have had ambivalent feelings towards whether to stay home that might have created stress. Alternatively, the fear of contagion as a result of those few occasions on which they went out might have also led to increased stress. Future studies might consider exploring this further by collecting qualitative data on individuals’ feelings and perceptions regarding the risk and stress of social engagement activities during the pandemic.

Aspects of our study design influence the interpretation of our study findings. First, as an observational cross-sectional study, there are issues with directionality of effects. Responses were collected at a single point in time, near the start of the semester, making it difficult to discern whether other unreported factors were associated with the selected social and behavioral activities or their mental health symptoms. Furthermore, we cannot determine whether increased social engagement led to reduced depressive symptoms or whether decreased social engagement was a consequence of those mental health symptoms. Aside from consuming alcohol, we are unaware of what additional in-person social exchanges occurred. Previous research found that very frequent in-person social connections during the pandemic were associated with lower depression [38].

Another limitation of this study was the use of a self-reported data collected via an online survey instrument. There is a possibility of bias due to under-reporting for some of our key selected variables. For instance, due to the legal age of tobacco and nicotine products having been raised to 21 matching the legal age of consumption for alcohol, there may have been some under-reporting of substance use of individuals age 18 and 20 [39]. However, self-response surveys for nicotine use and mental health symptoms are conventional for this type of research [40, 41]. Further, an additional limitation of our cross-sectional design of the study is the inability to capture the fluidity of mental health symptoms. Lastly, unlike in the Charles, Strong [24] study, there were no pre-pandemic matched mental health scores to compare changes in symptoms among our sample.

Strengths of this study includes the random sampling of the students, reducing the risk of sampling bias. However, it was vulnerable to volunteer bias. It is possible that eligible students who were experiencing extreme hardships were unable to participate in this study. Therefore, although our study findings might be representative of the general undergraduate student body, it might not be generalizable to some subgroups that were at severe risk of depression or stress.

During the regular college experience, college students experience a breadth of challenges, whether personal, academic, financial, or otherwise. The COVID-19 pandemic worsened some of those existing challenges and introduced new ones. Having conducted this study at the start of the Fall 2020 academic semester, as students returned from an all-online curriculum to a hybrid learning mode, is not representative of all the different stages of the pandemic. However, it offers some insight into students’ mental health and well-being for future public health crises.

**Conclusion**

These findings suggest that social engagement was associated with lower levels of depressive symptoms among college students during the fall 2020 semester. It may be that social engagement acted as a protective factor for depression during this time. Offering social activities that align with recommended safety precautions and meet students’ social needs should be considered as an important priority for higher education institutions as they continue to address the COVID-19 pandemic and plan for future public health emergencies. The mental health of students has been identified as a top concern for college and university leadership [42], and this study provides additional information about undergraduate mental health outcomes during the ongoing pandemic to inform policies, interventions, and the provision of services to address these issues.

**Acknowledgements**

**Code availability**

Not applicable.

**Ethics approval**

This study has been approved by the appropriate ethics committee and have therefore been performed in accordance with the ethical standards laid down in the 1964 Declaration of Helsinki and its later amendments.

**Consent to participate**

All participants enrolled in the study provided voluntary electronic written informed consent prior to their inclusion in the study.

**Consent for publication**

All participants enrolled in the study provided voluntary electronic written informed consent prior to their inclusion in the study, which included consent to the publication of anonymized data.

References

1.	American Psychological Association. Stress in America™ 2020: A National Mental Health Crisis. 2020.

2.	Jao NC, Robinson LD, Kelly PJ, Ciecierski CC, Hitsman B. Unhealthy behavior clustering and mental health status in United States college students. J Am Coll Health. 2019;67(8):790-800.

3.	Villarroel MA, Terlizzi EP. Symptoms of Depression Among Adults: United States, 2019. NCHS Data Brief. 2020(379):1-8.

4.	Larcombe W, Finch S, Sore R, Murray CM, Kentish S, Mulder RA, et al. Prevalence and socio-demographic correlates of psychological distress among students at an Australian university. Studies in Higher Education. 2016;41(6):1074-91.

5.	The Condition of Education. College Student Employment. 2020.

6.	Schulenberg JE, Johnston LD, O'Malley PM, Bachman JG, Miech RA, Patrick ME. Monitoring the Future: National Survey Results on Drug Use, 1975-2019: Volume II, College Students and Adults Ages 19-60. Ann Arbor: Institute for Social Research, The University of Michigan2020.

7.	Tafet GE, Bernardini R. Psychoneuroendocrinological links between chronic stress and depression. Progress in Neuropsychopharmacology & Biological Psychiatry. 2003;27(6):893-903.

8.	Wilson OWA, Holland KE, Elliott LD, Duffey M, Bopp M. The Impact of the COVID-19 Pandemic on US College Students' Physical Activity and Mental Health. Journal of Physical Activity & Health. 2021;18(3):272-8.

9.	Shinew KJ, Parry DC. Examining college student's participation in the leisure pursuits of drinking and illegal drug use. Journal of Leisure Research. 2005;37(3):364-86.

10.	Substance Abuse and Mental Health Services Administration. National Survey on Drug Use and Health, 2018 and 2019. Center for Behavioral Health Statistics and Quality; 2020.

11.	Bollen Z, Pabst A, Creupelandt C, Fontesse S, Lannoy S, Pinon N, et al. Prior drinking motives predict alcohol consumption during the COVID-19 lockdown: A cross-sectional online survey among Belgian college students. Addict Behav. 2021;115:106772.

12.	Choi BM, Abraham I. The Decline in e-Cigarette Use Among Youth in the United States-An Encouraging Trend but an Ongoing Public Health Challenge. JAMA Netw Open. 2021;4(6):e2112464.

13.	Gaiha SM, Lempert LK, Halpern-Felsher B. Underage Youth and Young Adult e-Cigarette Use and Access Before and During the Coronavirus Disease 2019 Pandemic. JAMA Netw Open. 2020;3(12):e2027572.

14.	Mak WW, Law RW, Woo J, Cheung FM, Lee D. Social support and psychological adjustment to SARS: the mediating role of self-care self-efficacy. Psychol Health. 2009;24(2):161-74.

15.	Galea S, Merchant RM, Lurie N. The Mental Health Consequences of COVID-19 and Physical Distancing: The Need for Prevention and Early Intervention. JAMA Internal Medicine. 2020;180(6):817-8.

16.	Haliwa I, Spalding R, Smith K, Chappell A, Strough J. Risk and protective factors for college students' psychological health during the COVID-19 pandemic. J Am Coll Health. 2021:1-5.

17.	Bu F, Steptoe A, Fancourt D. Who is lonely in lockdown? Cross-cohort analyses of predictors of loneliness before and during the COVID-19 pandemic. Public Health. 2020;186:31-4.

18.	Browning MHEM, Larson LR, Sharaievska I, Rigolon A, McAnirlin O, Mullenbach L, et al. Psychological impacts from COVID-19 among university students: Risk factors across seven states in the United States. PLoS One. 2021;16(1):e0245327.

19.	Yang C, Chen A, Chen Y. College students' stress and health in the COVID-19 pandemic: The role of academic workload, separation from school, and fears of contagion. PLoS One. 2021;16(2):e0246676.

20.	Cai G, Lin Y, Lu Y, He F, Morita K, Yamamoto T, et al. Behavioural responses and anxiety symptoms during the coronavirus disease 2019 (COVID-19) pandemic in Japan: A large scale cross-sectional study. J Psychiatr Res. 2021;136:296-305.

21.	Prete G, Fontanesi L, Porcelli P, Tommasi L. The Psychological Impact of COVID-19 in Italy: Worry Leads to Protective Behavior, but at the Cost of Anxiety. Front Psychol. 2020;11:566659.

22.	Fruehwirth JC, Biswas S, Perreira KM. The Covid-19 pandemic and mental health of first-year college students: Examining the effect of Covid-19 stressors using longitudinal data. PLoS One. 2021;16(3):e0247999.

23.	Hathaway ED, Peyer KL, Doyle KA. A first look at perceived stress in southeastern university students during the COVID-19 pandemic. J Am Coll Health. 2021:1-4.

24.	Charles NE, Strong SJ, Burns LC, Bullerjahn MR, Serafine KM. Increased mood disorder symptoms, perceived stress, and alcohol use among college students during the COVID-19 pandemic. Psychiatry Res. 2021;296:113706.

25.	Schwartz KD, Exner-Cortens D, McMorris CA, Makarenko E, Arnold P, Van Bavel M, et al. COVID-19 and Student Well-Being: Stress and Mental Health during Return-to-School. Canadian Journal of School Psychology. 2021;36(2):166-85.

26.	Harris PA, Taylor R, Minor BL, Elliott V, Fernandez M, O'Neal L, et al. The REDCap consortium: Building an international community of software platform partners. J Biomed Inform. 2019;95:103208.

27.	Kianersi S, Luetke M, Ludema C, Valenzuela A, Rosenberg M. Use of research electronic data capture (REDCap) in a COVID-19 randomized controlled trial: a practical example. BMC Med Res Methodol. 2021;21(1):175.

28.	Kianersi S, Ludema C, Macy JT, Garcia Colato E, Chen C, Luetke M, et al. A Cross-Sectional Analysis of Demographic and Behavioral Risk Factors of Severe Acute Respiratory Syndrome Coronavirus 2 Seropositivity Among a Sample of U.S. College Students. J Adolesc Health. 2021;69(2):219-26.

29.	Radloff LS. The CES-D Scale: A Self-Report Depression Scale for Research in the General Population. Appl Psychol Meas. 1977;1(3):385-401.

30.	Andresen EM, Malmgren JA, Carter WB, Patrick DL. Screening for depression in well older adults: Evaluation of a short form of the CES-D. Am J Prev Med. 1994;10(2):77-84.

31.	Zhang W, O'Brien N, Forrest JI, Salters KA, Patterson TL, Montaner JS, et al. Validating a shortened depression scale (10 item CES-D) among HIV-positive people in British Columbia, Canada. PLoS One. 2012;7(7):e40793.

32.	Cohen S, Kamarck T, Mermelstein R. A Global Measure of Perceived Stress. J Health Soc Behav. 1983;24(4):385-96.

33.	Swaminathan A, Gnanadurai T, Ayyavoo S, Viswanathan S, Manickam T. Perceived stress and sources of stress among first-year medical undergraduate students in a private medical college – Tamil Nadu. National Journal of Physiology, Pharmacy and Pharmacology. 2016;6(1):9-14.

34.	Kecojevic A, Basch CH, Sullivan M, Davi NK. The impact of the COVID-19 epidemic on mental health of undergraduate students in New Jersey, cross-sectional study. PLoS One. 2020;15(9):e0239696.

35.	Graves BS, Hall ME, Dias-Karch C, Haischer MH, Apter C. Gender differences in perceived stress and coping among college students. PLoS One. 2021;16(8):1-12.

36.	Hoyt LT, Cohen AK, Dull B, Maker Castro E, Yazdani N. “Constant Stress Has Become the New Normal”: Stress and Anxiety Inequalities Among U.S. College Students in the Time of COVID-19. J Adolesc Health. 2021;68(2):270-6.

37.	Obisesan OH, Mirbolouk M, Osei AD, Orimoloye OA, Uddin SMI, Dzaye O, et al. Association Between e-Cigarette Use and Depression in the Behavioral Risk Factor Surveillance System, 2016-2017. JAMA Network Open. 2019;2(12):e1916800.

38.	Rosenberg M, Luetke M, Hensel D, Kianersi S, Fu TC, Herbenick D. Depression and loneliness during April 2020 COVID-19 restrictions in the United States, and their associations with frequency of social and sexual connections. Soc Psychiatry Psychiatr Epidemiol. 2021;56(7):1221-32.

39.	U.S. Food and Drug Administration. Newly Signed Legislation Raises Federal Minimum Age of Sale of Tobacco Products to 21. 2020.

40.	Haeffel GJ, Howard GS. Self-report: psychology's four-letter word. Am J Psychol. 2010;123(2):181-8.

41.	Doran N, Correa JB, Myers MG, Tully L. Associations Between Self-Reported and Biological Measures of Nicotine Consumption Among Young Adult Nondaily Cigarette Smokers. Am J Addict. 2020;29(6):471-5.

42.	Turk JM, Soler MC, Ramos AM. College and university presidents respond to COVID-19: 2020 Fall term survey. American Council on Education; 2020.
